# Applications and Recruitment Performance of Web-Based Respondent-Driven Sampling: Scoping Review

**DOI:** 10.2196/17564

**Published:** 2021-01-15

**Authors:** Yannick B Helms, Nora Hamdiui, Mirjam E E Kretzschmar, Luis E C Rocha, Jim E van Steenbergen, Linus Bengtsson, Anna Thorson, Aura Timen, Mart L Stein

**Affiliations:** 1 National Coordination Centre for Communicable Disease Control Centre for Infectious Disease Control National Institute for Public Health and the Environment Bilthoven Netherlands; 2 Julius Center for Health Sciences and Primary Care University Medical Center Utrecht Utrecht Netherlands; 3 Department of Primary and Community Care Radboud University Medical Center Radboud Institute for Health Sciences Nijmegen Netherlands; 4 Department of Economics & Department of Physics and Astronomy Ghent University Ghent Belgium; 5 Centre for Infectious Diseases Leiden University Medical Centre Leiden Netherlands; 6 Flowminder Foundation Stockholm Sweden; 7 Department of Public Health Sciences Karolinska Institutet Stockholm Sweden; 8 Athena Institute for Research on Innovation and Communication in Health and Life Sciences Vrije Universiteit Amsterdam Amsterdam Netherlands

**Keywords:** respondent-driven sampling, webRDS, online sampling, public health, interventions, research methodology, hard-to-reach populations, probabilistic sampling

## Abstract

**Background:**

Web-based respondent-driven sampling is a novel sampling method for the recruitment of participants for generating population estimates, studying social network characteristics, and delivering health interventions. However, the application, barriers and facilitators, and recruitment performance of web-based respondent-driven sampling have not yet been systematically investigated.

**Objective:**

Our objectives were to provide an overview of published research using web-based respondent-driven sampling and to investigate factors related to the recruitment performance of web-based respondent-driven sampling.

**Methods:**

We conducted a scoping review on web-based respondent-driven sampling studies published between 2000 and 2019. We used the process evaluation of complex interventions framework to gain insights into how web-based respondent-driven sampling was implemented, what mechanisms of impact drove recruitment, what the role of context was in the study, and how these components together influenced the recruitment performance of web-based respondent-driven sampling.

**Results:**

We included 18 studies from 8 countries (high- and low-middle income countries), in which web-based respondent-driven sampling was used for making population estimates (n=12), studying social network characteristics (n=3), and delivering health-related interventions (n=3). Studies used web-based respondent-driven sampling to recruit between 19 and 3448 participants from a variety of target populations. Studies differed greatly in the number of seeds recruited, the proportion of successfully recruiting participants, the number of recruitment waves, the type of incentives offered to participants, and the duration of data collection. Studies that recruited relatively more seeds, through online platforms, and with less rigorous selection procedures reported relatively low percentages of successfully recruiting seeds. Studies that did not offer at least one guaranteed material incentive reported relatively fewer waves and lower percentages of successfully recruiting participants. The time of data collection was shortest in studies with university students.

**Conclusions:**

Web-based respondent-driven sampling can be successfully applied to recruit individuals for making population estimates, studying social network characteristics, and delivering health interventions. In general, seed and peer recruitment may be enhanced by rigorously selecting and motivating seeds, offering at least one guaranteed material incentive, and facilitating adequate recruitment options regarding the target population’s online connectedness and communication behavior. Potential trade-offs should be taken into account when implementing web-based respondent-driven sampling, such as having less opportunities to implement rigorous seed selection procedures when recruiting many seeds, as well as issues around online rather than physical participation, such as the risk of cheaters participating repeatedly.

## Introduction

Respondent-driven sampling (RDS) is a sampling method that leverages social networks for recruiting individuals from populations that lack a sampling frame. The method has often been used to sample hard-to-reach populations, such as men who have sex with men, people who use intravenous drugs, and individuals with a migration background [1,2].

RDS starts with a convenience sample of members of a target population. The initially recruited participants (known as *seeds*) then recruit individuals from their social network (known as *peers*). These recruitees, in turn, invite their own peers and so on, resulting in a series of waves of recruitment [2,3]. Usually, RDS utilizes a coupon system to track who recruits whom, and requires that participants self-report the size of their social network within the target population [3]. These data can be used in a statistical model to account for the nonrandom data collection. As such, under certain assumptions, RDS qualifies as a probability sampling method that can generate unbiased population estimates [4].

RDS has several applications besides generating population estimates. For example, data on links between individuals (obtained through tracking the recruitment process) allow for studying interactions within and between participants’ social networks. Among other things, this allows studying the spread of diseases in populations [5]. Furthermore, RDS can be used for recruitment of individuals for the delivery of health interventions [6,7].

Recruitment through RDS traditionally requires physical face-to-face interactions between individuals. However, over the past decade a novel online variant of RDS, so-called web-based RDS, was introduced. This potentially brings several benefits over offline RDS [8]. In particular, internet-based recruitment may (1) provide easy access and anonymity for participants; (2) overcome time- and location-related barriers to recruitment; and (3) provide an efficient, less laborious, and logistically demanding medium for recruitment from the researcher’s perspective [8-12]. However, web-based RDS also introduces challenges, such as selection bias resulting from differential access to the internet and problems with the credibility of online research [10].

Nevertheless, the application of web-based RDS, its potential benefits, and its drawbacks for recruiting individuals have not yet been studied. Therefore, we aimed to provide an overview of web-based RDS applications and to investigate factors related to its recruitment performance, by means of a scoping review. We are aware that the main purpose of typical RDS is to generate population estimations. However, since we focus on recruitment through web-based RDS, in this study, we were equally interested in reported experiences with using web-based RDS for the recruitment of individuals for the characterization of social networks and the delivery of interventions. We also highlight potential areas for future research on web-based RDS and formulate general recommendations for researchers interested in its application.

## Methods

### Study Design

A scoping literature review [13] was conducted to gain insights into the application and performance of web-based RDS. We chose to conduct a scoping review because our aims were primarily exploratory, in the sense that we intended to provide an overview of the work done with web-based RDS so far and to identify factors related to the recruitment performance of web-based RDS. Table 1 provides an overview of the web-based RDS terminology used in this review (partially adapted from [14]).

**Table 1:** Meaning of important web-based RDS terminology.

Terms	Meaning
Participant	An individual participating in a study or intervention.
Peers	A participant’s social contacts, such as friends or family members.
Coupon	An invitation (eg, in the form of a URL) that a participant can send to his/her peers, from the same target population, to invite peers in the study/intervention. Coupons use unique identifier codes to link recruiters with their recruitees.
Peer recruitment	The process of participants recruiting their peers.
Seed	A member of the target population who is recruited by a researcher to initiate peer recruitment.
Recruiter	A participant who recruits a peer by sending a coupon.
Recruitee	An individual who receives a coupon from a recruiter and agrees to enroll in the study/intervention.
Recruitment tree	A visualization of the peer recruitment process, in which all recruiters and their recruitees are linked in chains.
Wave	The distance (the number of chain-links) between seeds and their recruitees, in which seeds are in wave 0, their recruitees in wave 1, and so on.
Equilibrium	Equilibrium is reached when the sample composition of selected key indicators (eg, age and gender) remains stable over successive waves. Equilibrium indicates that the sample has become independent of the initially selected seeds.
Recruitment options	The options that participants have to forward their coupons to their peers.
Incentive	The stimuli provided to an individual for participation (primary incentive) or for each individual recruited (secondary incentive) to stimulate peer recruitment. An incentive can be material (tangible, eg, a gift card) or nonmaterial (intangible, eg, anonymous survey results).
Incentive structure	If only a primary or secondary incentive is offered, this is referred to as a single incentive structure; if both are offered, this is referred to as a double incentive structure.
Recruitment performance measures	Measures for recruitment performance (eg, number of individuals recruited) used in this research.

### Search Strategy and Article Selection

We searched PubMed, Web of Science, and Scopus for articles. First, a preliminary search was conducted in PubMed to gauge the quality and quantity of web-based RDS related articles and to identify keywords to formulate the search syntaxes.

The term *web-based RDS* was introduced in 2008. In order to ensure that potentially relevant articles from before the term was introduced were included, we set our search range as 2000 to 2019. The following search terms were included in the final search syntaxes (see Multimedia Appendix 1 for the full syntaxes used):

1. Study type: implementation, development, testing, adoption, pilot
2. Online: online, web-based, internet, internet-based
3. Recruitment strategy: respondent-driven, peer-driven, participant-driven, snowball, chain-referral
4. Study purpose: intervention, sampling, recruitment, referral

Two researchers (YH and MS) independently screened the titles and abstracts of all unique records identified. The full text of selected records were then screened by one researcher (YH), to apply the below eligibility criteria. After this, the remaining articles were critically reviewed by YH, MS, and NH, before being included.

### Eligibility Criteria

We included peer-reviewed articles that described the use of web-based RDS for the recruitment of participants for research purposes (ie, for making population estimates or for studying social networks) or for health intervention delivery. Articles that at least reported the numbers of seeds, subsequent recruitees, and discussed barriers and facilitators to the application of web-based RDS were included. We excluded studies that combined online and offline RDS without reporting on both approaches separately; if a study reported online and offline RDS separately, the online component was included. As we meant to provide an overview of the applications and performance of web-based RDS, our study was not limited to any particular target population or geographical area. We excluded studies that were not available in English or Dutch.

### Data Extraction and Analysis

A data extraction table was developed to collect and organize data. The table’s topics were iteratively identified and selected based on (1) STROBE (Strengthening the Reporting of Observational Studies in Epidemiology) RDS guidelines [15], (2) topics discussed in a literature review with a similar purpose and context (offline RDS) to this study [1], and (3) discussion between YH, MS, and NH. Additional topics included study design, main findings, recommendations for further research, and limitations.

We used the process evaluation of complex interventions framework [16] to analyze the application and recruitment performance of web-based RDS. This framework explains the outcomes of an intervention as a function of implementation characteristics, mechanisms of impact, and contextual factors. We adapted this framework to fit web-based RDS specifications. In this study, we viewed web-based RDS as an intervention with the purpose of recruiting individuals. We defined outcomes as web-based RDS recruitment performance, implementation characteristics as the seed selection and recruitment process, mechanisms of impact as mechanisms to stimulate peer recruitment, and context as the setting in which web-based RDS was conducted.

**Figure 1:**
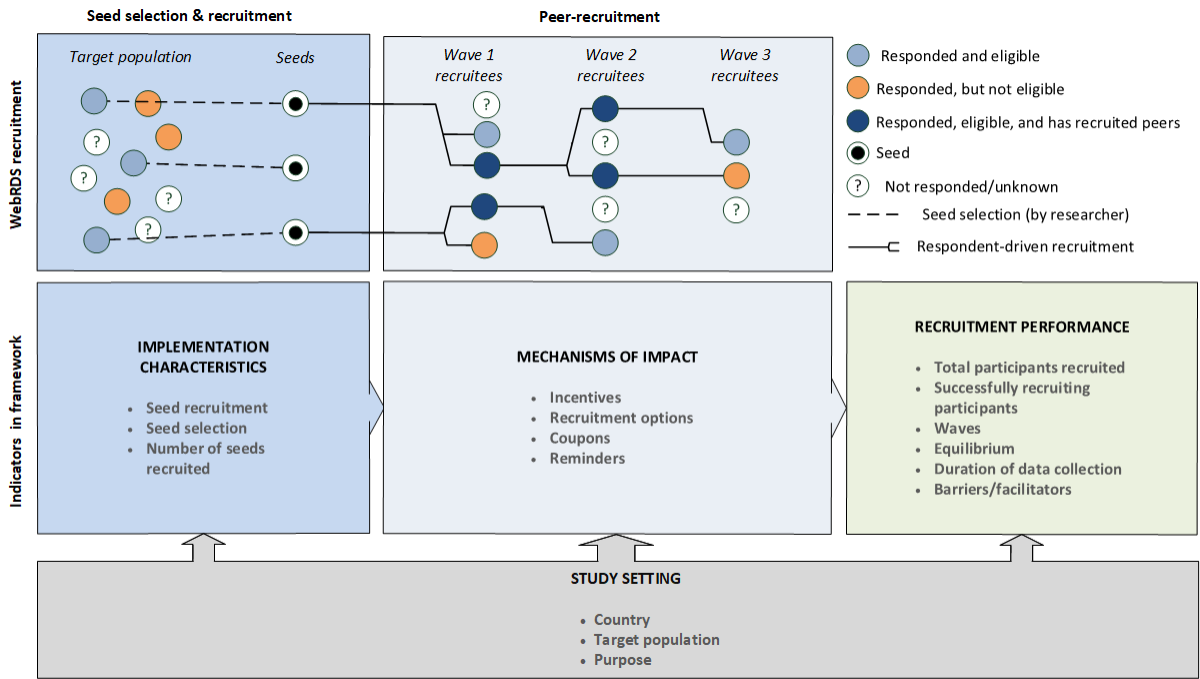
Analytical framework for web-based RDS recruitment performance (adapted from [16]).

Figure 1 (adapted from [16]) shows the analytical framework integrated with a schematic representation of the web-based RDS recruitment process. Table 2 shows the extraction table’s topics, and operationalized measures thereof, grouped by components of the process evaluation framework.

Analyses focused on uncovering factors that influenced recruitment performance, based on comparing implementation characteristics, peer recruitment and recruitment performance measures between included studies. Data were presented in a narrative fashion.

**Table 2:** Topics for data extraction and associated measures.

Topics		Measures
**Study setting (context)**		
	Country	Country
	Target population	Target population (specify)
	Purpose	Study purpose (eg, population estimates, social networks, intervention)
**Seed recruitment and selection (implementation characteristics)**		
	Seed recruitment	Recruitment platform (specify)
	Seed selection	Selection procedure (specify)
	Number of seeds recruited	Number of seeds recruited that participated in the study
**Peer recruitment (mechanisms of impact)**		
	Incentives	Material or nonmaterial (specify) Single or double (specify) Maximum value of incentive
	Recruitment options	Recruitment options (specify)
	Coupons	Number of coupons allowed (ie, that a participant can forward)
	Reminders	Reminders sent to enhance recruitment (yes/no)
**Recruitment performance (outcomes)**		
	Total participants recruited	Sample size^a^
	Successfully recruiting participants^b,c^	Proportion of seeds who successfully initiated recruitment Proportion of all participants who successfully recruited peers to the study
	Waves	Maximum number of waves observed
	Equilibrium	Equilibrium reached (yes/no, and after how many waves)
	Duration of data collection	Duration of data collection
	Barriers and facilitators	Barriers and facilitators indicated to have influenced recruitment performance

^a^Sample size was calculated as sample size minus duplicate or fraudulent entries, if reported.

^b^We defined a successfully recruiting participant as a participant who invites at least one other person who participates in the study, regardless of the eligibility (the latter says more about how strict or elaborate researchers set eligibility criteria rather than about participants’ ability to peer recruit). This excludes participants who merely sent out invitations with no response. If reported, duplicate or fraudulent entries were excluded.

^c^If not otherwise reported, this metric was manually counted and calculated from the reported recruitment tree.

### Ethical Considerations

No ethical issues were foreseen in this study.

## Results

### Included Studies

**Figure 2:**
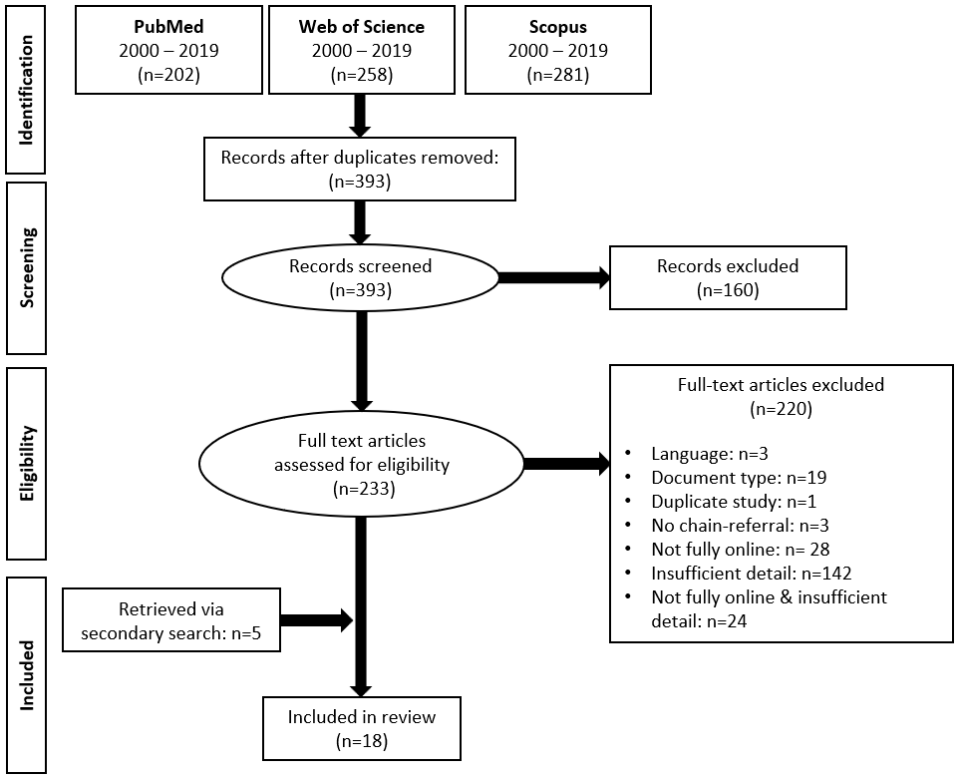
PRISMA flowchart.

We identified 393 unique records. The final number of articles included in this review was 18. See Figure 2 for a detailed account of the study inclusion procedure.

### Study Settings

Six studies took place in the United States [8,17-21], 6 studies took place in Western Europe [22-27], 4 studies took place in southeast Asia [23,28-31], and 2 studies took place in Oceania [32,33]. Studies were published between 2008 and 2019, with the majority [17,18,22-24,26,27,30,32,33] from 2015 onward (see Table 3).

In 12 studies, participants were recruited with the aim of generating population estimates [8,19-23,26,28,29,31-33]; 3 studies aimed to study social networks and contact patterns relevant to the spread of infectious diseases [24,25,28], and 3 studies recruited participants for delivering interventions [17,18,27].

Studies focused on a diverse set of target populations: the general population [19,24,25,28,30,32], university students [8,20], men who have sex with men [23,29,31], individuals who smoke [17], individuals using wheelchairs [33], parents of children aged 10 to 14 [18], people with precarious employment [22], young adults at risk of chlamydia infection [27], individuals who have migrated from Syria [26], and individuals who use marijuana [21].

**Table 3:** Characteristics of the articles included in review.

Reference	Setting	Study purpose	Target population	Final sample size (excluding fraudulent/duplicate entries, as reported)
Jonsson et al [22]	Stockholm county, Sweden	Generating population estimates	People with precarious employment	595 (551)
Weinmann et al [26]	Munich, Germany	Generating population estimates	Individuals who have migrated from Syria	195 (—^a^)
Oesterle et al [18]	Washington and Colorado, United States	Intervention delivery	Parents of children in grades 6-8 (ie, aged 10-14 years)	235 (—)
Tran et al [30]	Vietnam	Generating population estimates	General population (youth and young adults)	366 (356)
Sadasivam et al [17]	United States	Intervention delivery	Individuals who smoke	759 (—)
Bourke et al [33]	New Zealand	Generating population estimates	Individuals using wheelchairs	19 (19)
Hildebrand et al [32]	Perth metropolitan area, Australia	Generating population estimates	General population (youth)	780 (—)
Stein et al [24]	Netherlands	Studying social networks and contact patterns relevant to the spread of infectious diseases	General population	1448 (1429)
Stromdahl et al [23]	Sweden	Generating population estimates	Men who have sex with men	148 (130)
Theunissen et al [27]	South Limburg, Netherlands	Intervention delivery	Young adults at risk of a *Chlamydia trachomatis* infection	68 (68)
Bengtsson et al [31]	Vietnam	Generating population estimates	Men who have sex with men	982 (870)
Crawford [21]	Oregon, United States	Generating population estimates	Individuals who use marijuana	72 (—)
Stein et al [25]	Netherlands and Thailand	Studying social networks & contact patterns relevant to the spread of infectious diseases	General population	358 (—)
Stein et al [28]	Thailand	Studying social networks & contact patterns relevant to the spread of infectious diseases	General population	257 (245)
Bauermeister et al [19]	United States	Generating population estimates	General population (young adults)	3448 (—)
Bengtsson et al [29]	Vietnam	Generating population estimates	Men who have sex with men	676 (591)
Wejnert [20]	Cornell University, United States	Generating population estimates	University students	378 (—)
Heckathorn and Wejnert [8]	Cornell University, United States	Generating population estimates	University students	159 (—)

^a^Not specified.

### Recruitment-Related Results

#### Seed Recruitment and Selection

Five studies recruited seeds through online platforms in the form of targeted Facebook advertisements [17-19,32] or online participatory research panels [24] (see Multimedia Appendix 2), while 6 studies combined online (eg, online advertisements) with offline platforms, such as interest groups [23,26,28], researchers’ social networks [22,25,26,28], or social venues [22,26,29,31] and 2 studies only recruited seeds offline—1 at a sexual health clinic [27] and 1 through a previous research project [21].

In most studies, researchers established contact with potential seeds as part of the seed selection procedure. This was done to confirm potential seeds’ identity or eligibility [22,27,32,33], to select seeds with specific characteristics [19,22,26,30], or to confirm potential seeds’ motivation and ability to invite and recruit peers [21-23,30]. Contact between researchers and seeds consisted of phone calls [19,22], emails [8,18,25,28], or in-person or group meetings [25,27,28]. In 3 studies, seed selection consisted only of an online eligibility screener and consent form [17,24,30].

The number of recruited seeds ranged between 1 [21] and 1015 [24].

#### Peer Recruitment

Most studies allowed participants to recruit their peers (eg, by sharing a URL) through preferred means of communication, such as WhatsApp or Facebook [17,19,21,22,29-32] or email [8,20,33]. Some studies additionally provided participants with the opportunity to provide their peers’ contact details to the researchers, after which they contacted participants’ peers via email (see Multimedia Appendix 2) [22,24,25,28,29,31].

In 4 studies, there was no limit for the number of coupons that participants could forward [17,18,27,30]. In other studies, the limit was 3 coupons [8,20,26,32,33], 4 coupons [22-25,28,29,31], 5 coupons [21], or 10 coupons [19].

Most studies had a double-incentive structure [8,17-20,22,23,26,29,31-33]. The majority of studies used material incentives as opposed to [8,19,20,22,23,26,30,32,33] or in combination with [17,18,24,29,31] nonmaterial incentives, and 4 studies only used nonmaterial incentives [21,25,27,28].

Material incentives were electronic gift cards [19,22,23,26,32], phone credits [29-31], or lottery tickets [24,29,31,33]. Nonmaterial incentives included showing participants aggregated study results [21,29,31] or visual insights into the study’s recruitment process (eg, anonymized recruitment trees) [17,21,24,25,28], allowing participants to donate material incentives [29,31], and delivering political or social messages to prospective participants [18,21].

The maximum value of material incentives that participants could earn ranged between US $12.45 in Vietnam [29,31] and US $115 in the United States [17], but 2 studies had no maximum incentive value, since their number of incentivized coupons was unlimited [18,30].

Nine studies reported having sent reminders [17,22,24,27-29,31-33].

#### Recruitment Performance

The final sample sizes ranged between 19 [33] and 3448 [19]. Three studies recruited less than 100 participants [21,27,33], and 6 studies recruited more than 600 participants [17,19,24,29,31,32] (see Multimedia Appendix 2).

The proportion of seeds and the proportion of all participants who successfully recruited ranged between 7.5% [18] and 100% [21], and between 9.2% [27] and 55% [8], respectively. The maximum number of waves ranged between 1 [33] to 29 [31], and 8 studies reported fewer than 10 waves [17,21,23-25,27,28,33]. RDS sample distribution reached equilibrium in 5 studies, after 1 to 11 waves [8,20,22,26,29,31].

Data collection took between 72 hours [8] and 7 months [25]. In most studies, data collection took between 2 months and 3 months [18-21,26,28,30].

#### Factors Influencing Recruitment Performance

Overall, studies that recruited more seeds relatively often used online platforms (such as Facebook or other web communities) for seed recruitment, selected seeds less rigorously (eg, with less or no contact between potential seeds and researchers), and recruited seeds from larger geographical areas (eg, the entire country as opposed to a municipal area). Studies that recruited fewer seeds relatively often did so through a combination of both online and offline, or only offline platforms, with more elaborate seed selection procedures, and in smaller geographical areas.

Studies that recruited more seeds, through online platforms, and with less rigorous selection procedures, reported relatively lower percentages of successfully recruiting seeds. For example, the 3 studies that recruited the most seeds reported between 7.6% and 24.7% successfully recruiting seeds [17,18,24], compared to 67.4% to 100% in the 3 studies with the fewest recruited seeds [8,20,21].

Studies that did not offer at least one guaranteed material incentive (ie, not lottery-based compensation) reached no more than 6 waves and reported between 9.2% and 38.9% successfully recruiting participants [21,25,27,28].

Authors of included studies suggested a lack of monetary incentives [21,25,28,33], a lack of different recruitment options [19,22], and cheating (in order to retrieve multiple incentives) by participants [8,19,29,32] as potential factors related to web-based RDS protocols that influenced recruitment performance. The incompatibility of questionnaires or recruitment options with mobile platforms [18,19,23,30], and recruitment emails being identified as spam [8,19,28] were suggested as technical difficulties influencing recruitment performance. Seeds’ motivation (or a lack thereof) to initiate recruitment [22,28,32,33], stigma regarding the study subject [27,33], online connectedness of the target population [8,17,22,26,33], and differential access to the Internet [8,18,19,28-32], were suggested as psychological and structural characteristics of the target population influencing recruitment performance.

## Discussion

### Overview

This is the first review investigating the application and recruitment performance of web-based RDS; a novel online sampling method. We identified 18 articles that described the use of web-based RDS. Out of all studies, 12 recruited participants for making population estimates, 3 recruited participants to study social network characteristics (contact patterns relevant to the spread of infectious diseases), and 3 recruited participants to deliver interventions. Studies were conducted in 8 countries, including both high- and low-middle income countries, over 4 continents. Between 19 and 3448 participants were recruited from various populations, including some without a sampling frame, such as men who have sex with men. The heterogenous nature of the included studies (with respect to their aims and setup) made it difficult to compare their recruitment processes and to draw generalizable inferences regarding recruitment performance.

### Principal Findings

We found that studies that recruited relatively more seeds, through online platforms, and with less rigorous selection procedures reported lower percentages of successfully recruiting seeds. The exact reasons for this observation remain unclear. However, we suggest that recruiting more seeds relatively limits the time and resources available to thoroughly prepare (ie, motivate and inform) and select seeds. In turn, this may limit seeds’ motivation to initiate peer recruitment, or lead to less suited (eg, less socially connected) seeds being selected. Both of these factors are known to be important for inducing and sustaining seed and peer recruitment [34,35].

Studies that did not offer at least one guaranteed material incentive reached relatively lower percentages of successfully recruiting participant, and fewer waves. We thus suggest that such incentives are particularly important to sustain recruitment, as sampling waves increase (ie, monetary incentives appear to carry further than nonmonetary incentives). This is in agreement with wider offline RDS literature [34] and indicates that benefits of online recruitment for participants (eg, easy access for participants) and nonmonetary incentives do not render material incentives redundant if the primary aim is to generate recruitment waves.

However, some studies that recruited participants for interventions reported relatively low percentages of successfully recruiting participants, despite offering substantial monetary incentives. This indicates that online peer recruitment for interventions benefits (or suffers) from factors other than peer recruitment for research purposes. Potentially, peer recruitment for interventions depends more on participants’ affinity toward an intervention (eg, related to intervention framing, packaging), or its anticipated or experienced outcomes. Note, however, that these findings are based only on few studies and require further research to substantiate.

The majority of studies took between 2 and 3 months to collect data. However, online recruitment was relatively faster in some studies. For example, one study [8] achieved their targeted sample size (N=150) in 72 hours, and another [19] recruited 3448 participants in 6 weeks. Both these study populations were composed of university students who may be particularly digitally literate and have extensive well-connected online networks. This finding indicates the importance of these factors when considering applying this online method.

### Strengths and Limitations

One strength of this literature review was the wide search strategy. It provides an extensive overview of peer reviewed literature relevant for investigating web-based RDS peer-recruitment processes. Another strength was the application of the process evaluation framework, which offered a practical structure for investigating different factors influencing web-based RDS recruitment performance.

One limitation is that we excluded all articles not reporting on the recruitment process in sufficient detail and all studies not exclusively using online peer recruitment or reporting on online and offline peer recruitment separately. Some valuable contextual and comparative information between online and offline recruitment might therefore have been missed. For some crucial recruitment performance measures (eg, the percentage of successfully recruiting participants), we had to rely on a manual count of recruitment trees, since the original data sets were unavailable.

### Practical Implications and Opportunities for Future Research Using Web-Based RDS

Based on this review, it remains difficult to assess how successful web-based RDS is at achieving the aims for which it is employed (ie, generating population estimates, studying social networks, delivering interventions). For example, only 5 out of 12 studies aiming to generate population estimates reported that the sample composition reached equilibrium. Several studies likely achieved equilibrium (estimated from reported sample size and observed number of waves), but did not report this as such. Studies that used web-based RDS for studying social networks or delivering interventions were mostly feasibility or implementation studies, making it difficult to assess how successful the online method is at reaching the endpoints. At this point in time, we believe that there are not enough studies to draw meaningful conclusions regarding the overall success of web-based RDS for generating population estimates, studying social networks, or delivering interventions. Nevertheless, web-based RDS may be a particularly suited recruitment method when random sampling techniques are not feasible, the target population is geographically dispersed or hidden (which is a challenge for offline sampling), and the target population is extensively connected online [8]. Therefore, despite the heterogenous nature of the studies included in this review, which limits the generalizability of the studies’ recruitment processes and performance, we outline several recommendations for future research into, or using web-based RDS.

First, consistent with offline RDS literature, the results suggest that recruiting a relatively small and thoroughly selected group of seeds (to whom a significant amount of resources can be dedicated for motivational and informing purposes) and providing at least one guaranteed material incentive is the most successful strategy for generating a substantial number of waves [1,34]. As such, this is the preferred setup for studies aiming to reach equilibrium for population estimates. If this is not the primary objective, for example when recruiting individuals for studying network characteristics or delivering interventions, recruiting a larger number of seeds through less rigorous means and providing lower or nonmaterial incentives may be preferred.

Second, despite the limited number of studies that recruited participants for interventions in this review, some implications in this regard stand out. For example, one study [27] found that through web-based RDS, individuals could be reached for sexually transmitted disease testing who were not reached before through traditional sexual health services. Another study [17] similarly noted that with each successive wave, the proportion of not-ready-to-quit smokers in the sample increased. These findings indicate that web-based RDS recruitment is particularly interesting for interventions if the aim is to reach more reluctant, or previously unreached individuals. The challenge here is to adequately incentivize peer recruitment (as discussed under principal findings).

Third, the results indicate that providing multiple recruitment options and facilitating the use of mobile platforms for participation and recruitment may enhance web-based RDS recruitment performance. However, it remains largely unclear how these factors influence peer recruitment across different settings, or even within certain target populations. For example, as indicated also by several studies included in this review, differential access to mobile communications, or the internet in general, may impose barriers to peer recruitment to readily excluded members of a given population. In addition, online communication behavior and the types of digital communication platforms used may differ between different networks, which could affect even relatively well-connected individuals. This is exemplified by one study [32] included in this review that compared web-based RDS to traditional offline RDS and found that individuals with lower socioeconomic status were less likely to be recruited through web-based RDS. Besides socioeconomic status, other factors known from literature that influence access to or use of the internet (and may therefore also influence online peer recruitment) include sociodemographic (eg, age, gender), socioeconomic (eg, household income, educational attainment), social (eg, degree of isolation, political context), and personal (eg, self-efficacy, mental health) factors [36]. To account for these potential sources of bias, we suggest thorough exploration of the target population’s online networks and communication behaviors, in a formative research stage.

Finally, we recommend that researchers using web-based RDS follow STROBE-RDS guidelines when reporting their studies [15]. A number of studies did not consistently report the numbers of total distributed and returned coupons, the numbers of recruitment waves, the numbers of individuals collecting their incentives, and the numbers of recruitees by seeds. Similar gaps in reporting on offline RDS data have been found in a previous review [37]. In addition, we encourage researchers to report relevant recruitment performance measures, such as the percentages of successful recruiters or the average numbers of recruitees per participant. This information is crucial for studying how to optimize peer recruitment in the future.

### Conclusions

We have given a comprehensive overview of web-based RDS, what it is used for, how it is applied, and what factors influence its recruitment performance. Web-based RDS can be successfully applied to recruit individuals for making population estimates, studying social networks, and delivering health interventions. Peer recruitment may be enhanced by rigorously selecting and motivating seeds, offering at least one guaranteed material incentive, and facilitating adequate recruitment options regarding target populations’ online connectedness and communication behavior. Potential trade-offs should be taken into account when implementing web-based RDS. Examples are recruiting many seeds and less opportunities for rigorous seed selection procedures, as well as issues around online rather than physical participation, such as risks of cheaters through repeated participation. Furthermore, we have demonstrated the feasibility of—and described outcome measures for—research into web-based RDS recruitment using a process evaluation approach. The main points discussed in this literature review provide researchers with guidelines on key aspects and technicalities to consider when planning their web-based RDS research.

## Appendix

Multimedia Appendix 1 Search syntaxes.

Multimedia Appendix 2 Recruitment results.

## Abbreviations

RDS: respondent-driven sampling
STROBE: Strengthening the Reporting of Observational Studies in Epidemiology
