# Supplementary material for: Applications and Recruitment Performance of Web-Based Respondent-Driven Sampling: Scoping Review
Source: J Med Internet Res. 2021 Jan 15;23(1):e17564. doi: 10.2196/17564 (PMC7846441; doi:10.2196/17564)
Supplement: Multimedia Appendix 1 [file jmir_v23i1e17564_app1.pdf]

Multimedia Appendix 1. Search syntaxes

Pubmed: (implement* OR develop* OR test* OR adopt* OR pilot*) AND (online OR “web based” OR “web-based” OR internet OR “internet based” OR “internet-based”) AND (“respondent driven” OR “respondent-driven” OR “peer driven” OR “peer-driven” OR “participant driven” OR “participant-driven” OR snowball OR “chain-referral” OR “chain-referral”) AND (intervention OR sampling OR recruitment OR referral)

Scopus: TITLE-ABS-KEY ((implement* OR develop* OR test* OR adopt* OR pilot*) AND (online OR “web based” OR “web-based” OR internet OR “internet based” OR “internet-based”) AND (“respondent driven” OR “respondent-driven” OR “peer driven” OR “peer-driven” OR “participant driven” OR “participant-driven” OR snowball OR “chain-referral” OR “chain-referral”) AND (intervention OR sampling OR recruitment OR referral))

Web of Science: TS=((implement* OR develop* OR test* OR adopt* OR pilot*) AND (online OR “web based” OR “web-based” OR internet OR “internet based” OR “internet-based”) AND (“respondent driven” OR “respondent-driven” OR “peer driven” OR “peer-driven” OR “participant driven” OR “participant-driven” OR snowball OR “chain-referral” OR “chain-referral”) AND (intervention OR sampling OR recruitment OR referral)
