# Supplementary material for: Applications and Recruitment Performance of Web-Based Respondent-Driven Sampling: Scoping Review
Source: J Med Internet Res. 2021 Jan 15;23(1):e17564. doi: 10.2196/17564 (PMC7846441; doi:10.2196/17564)
Supplement: Multimedia Appendix 2 [file jmir_v23i1e17564_app2.pdf]

Multimedia Appendix 2. Recruitment results

Reference	Seed recruitment and selection			Peer-recruitment						Recruitment performance				
	Seed recruitment platform	Seed selection	Nr. of seeds recruited	Incentives			Recruitment options	Max nr. of coupons allowed	Reminder sent Yes/No (freq.)	% of seeds successfully recruiting	% of all participants successfully recruiting	Max. nr. of waves	Final sample size (corrected [b])	Equilibrium (after N^{th} wave)
				Type	Structure	Max. value incentive								
Jonsson et at., 2019	On/offline	Phone call	57	Material	Double	$20	Email, Sharing web-link through preferred means of communication)	4	Yes	59.6[a]	49.6 [a]	15	595 (551)	Yes (n/s)
Weinmann et al., 2019	On/offline	n/s	16	Material	Double	$22 (E 20)	n/s	3	n/s	n/s	n/s	n/s	195 (n/s)	Yes (1)
Oesterle et al., 2018	Online	Online form and email or phone call	185	Material + non-material	Double	No limit	n/s	No limit	n/s	7.5	n/s	n/s	235 (n/s)	n/s
Tran et al., 2018	n/s	Online form	30	Material	Single	No limit	Sharing web-link through preferred means of communication	No limit	n/s	n/s	n/s	n/s	366 (356)	No
Sadasivam et al, 2017	Online	Online form	190	Material + non-material	Double	$115	Sharing web-link through preferred means of communication	No limit	Yes	24.7	15	4	759 (n/s)	n/s
Bourke et al., 2016	n/s	n/s	12	Material	Double	iPad (lottery-based)	Email	3	Yes	33	21	1	19 (19)	No
Hildebrand et al., 2015	On/offline(flyer)	n/s	68	Material	Double	$32 (AU$45)	Sharing web-link through preferred means of communication	3	Yes	n/s	n/s	12	780 (n/s)	n/s
Stein, M L et al, 2015	Online	Online form	1015	Material + non-material	Single	$28 (E25, lottery based)	Email, private Facebook messages	4	Yes	19.1	22.7	6	1448 (1429)	n/s
Stromdahl et al., 2015	On/offline	n/s	37	Material	Double	$20	Email, sharing web-link through preferred means of communication	4	n/s	n/s	35.1 [a]	9	148 (130)	No
Theunissen et al., 2015	Offline	In-person	30	Non-material	Single	None	Email	No limit	Yes	20 [a]	9.2 [a]	2	68 (68)	n/s
Bengtsson et al., (2014)	On/offline	n/s	13	Material + non-material	Double	$12.45 + iPad (lottery based)	Email, Yahoo Messenger, sharing web-link through preferred means of communication	4	Yes	76.9[a]	42.5[a]	29	982 (870)	Yes (11)
Crawford, 2014	Offline	n/s	1	Non-material	Single	None	Sharing web-link through preferred means of communication	5	n/s	100	38.9 [a]	5	72 (n/s)	No

Stein et al., 2014a	On/offline	In-group meetings and invitation email	92	Non-material	Single	None	Email, private Facebook message	4	n/s	72.8 [a]	38.8 [a]	5	358 (n/s)	n/s
Stein et al., 2014b	On/offline	In-group meetings and invitation email	89	Non-material	Single	None	Email, private Facebook message	4	Yes	49.4 [a]	33.9 [a]	6	257 (245)	No
Bauermeister et al., 2012	Online	Online form and Phone call	22	Material	Double	$70	Email, sharing web-link through preferred means of communication	10	n/s	n/s	n/s	n/s	3448 (n/s)	n/s
Bengtsson et al., 2012	On/offline	n/s	20	Material + non-material	Double	$12.45 + iPad (lottery based)	Email, Yahoo Messenger, sharing web-link through preferred means of communication	4	Yes	90 [a]	44.5 [a]	24	676 (591)	Yes (7)
Wejnert, 2009	n/s	n/s	9	Material	Double	$25	Email	3	n/s	77.8 [a]	51 [a]	23	378 (n/s)	Yes (9)
Wejnert & Heckathorn, 2008	n/s	n/s	9	Material	Double	$55	Email	3	n/s	67.4 [a]	55 [a]	18	159 (n/s)	Yes (9)

Abbreviation: n/s, not specified

[a] Counted from reported recruitment tree

[b] Corrected for cheating/duplicate entries if reported
